# Panel data to investigate pricing behavior in the Spanish retail fuel market^☆^

**DOI:** 10.1016/j.dib.2019.104880

**Published:** 2019-11-27

**Authors:** Xulia González, MaríaJ. Moral

**Affiliations:** a University of Vigo, Spain; b UNED, Spain

**Keywords:** Diesel prices, Retail fuel market, Brand affiliation, Panel data, Location

## Abstract

The data described in this article were collected daily over the period 18 August 2014 to 15 June 2015 from the website of the Spanish Ministry of Industry, Energy and Tourism http://geoportalgasolineras.es. The database includes information on almost all gas stations located in Spain that sell to the public. For each gas station we have information of daily diesel prices (both posted price and net of taxes), brands and locations (latitude and longitude) and the Brent price. We also share a Stata program file to identify the nearest competitors of each gas station and their brand. The program also computes the distance to the nearest refinery and its brand. The data base can be used for analyzing firms pricing behavior focusing, for example, on asymmetric pricing, cartels or vertical integration, among other topics. This data base is used in the paper [2] “Effects of antitrust prosecution on retail fuel prices” that analyze the impact on prices of an antitrust sanction imposed to several brands on February 2015.

**Table:** Specifications Table

Subject	Economics and Econometrics
Specific subject area	Energy Economics, Empirical Industrial Organization
Type of data	Microdata Panel data
How data were acquired	Data was daily extracted from the web site of Spanish Ministry of Industry, Energy and Tourism: http://geoportalgasolineras.es
Data format	Stata data set, CSV
Parameters for data collection	Data were collected daily from 18 August 2014 to 15 June 2015.
Description of data collection	The data include information about retail prices, pre-tax retail prices, brand of the gas station, geocoordinates (longitude and latitude) of the gas stations and the Spanish refineries. From the raw data we excluded gas stations that do not sell to the public, and those located in Canary and Balearic Islands. We also exclude the refinery located in Tenerife. The data set contains information of 8080 gas stations, and 1,888,507 price quotes.
Data source location	Spain
Data accessibility	Gonzalez, Xulia; Moral, María J. (2019), “panel_gas_stations ”, Mendeley Data. https://data.mendeley.com/datasets/jk9tnbpp3z/2
Related research article	Authors: Xulia González and María J. Moral Title: Effects of antitrust prosecution on retail fuel prices Journal: International Journal of Industrial Organization DOI: https://doi.org/10.1016/j.ijindorg.2019.102537

**Table:** 

**Value of the Data** • The panel data let to investigate pricing behavior in the Spanish retail fuel market. • Researchers in empirical industrial organization or energy economics can benefit from these data. • Analysis of brand heterogeneity in pricing strategies. • Analysis of cost pass-through in fuel markets. • Analysis of the effect of local market competition on retail fuel prices.

## 1 Data

The datasets contain raw data on daily diesel and Brent prices as well as some characteristics of 8080 gas stations located in Spain.

**Fig. 1:**
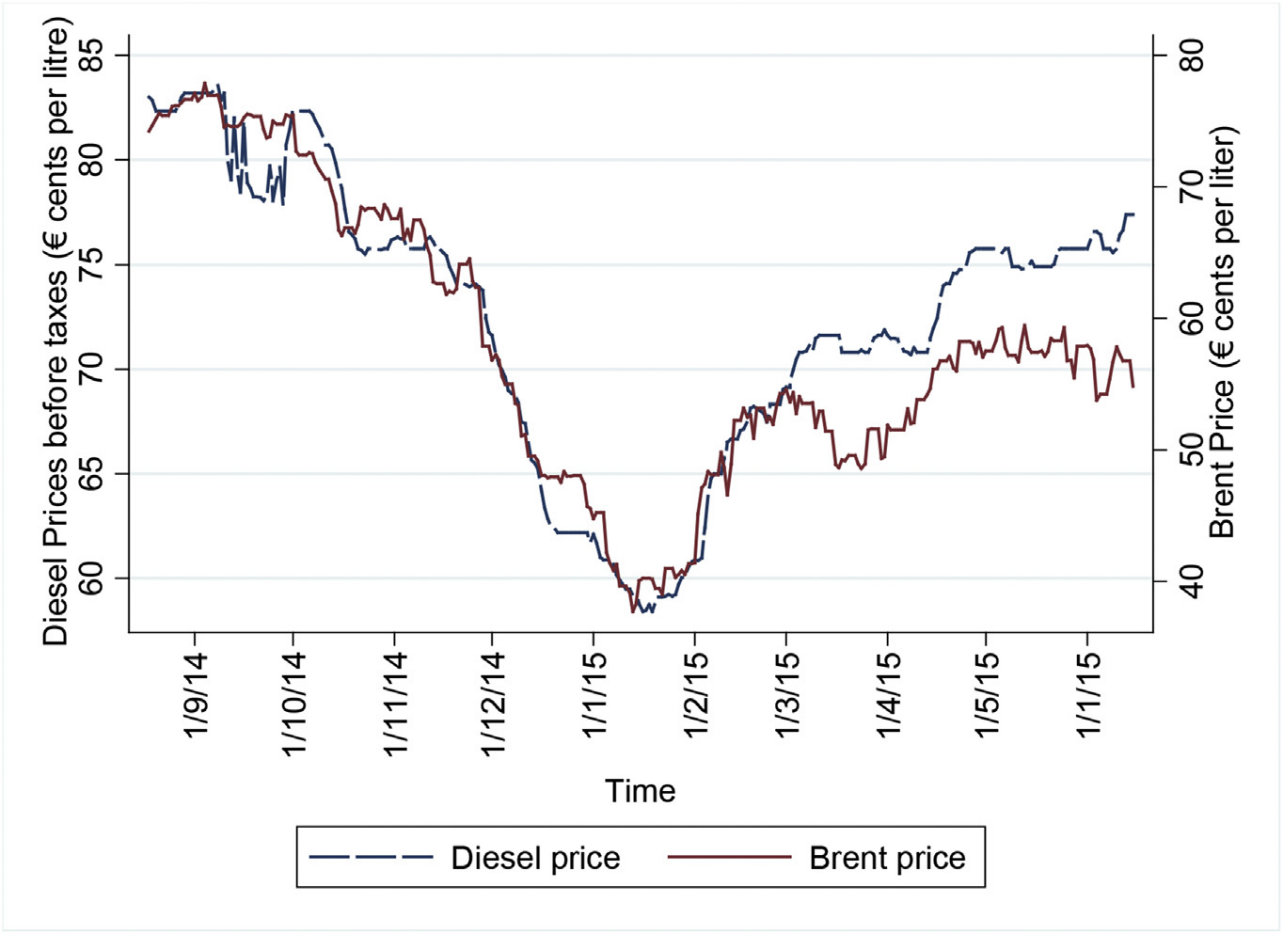
Daily Brent price and median value of diesel price (before taxes).

We share two data sets ready to read in CSV (and STATA). The first one (*panel_gas.csv*) is a panel data that contains information of daily prices (from August 18th^,^ 2014 to June 15th^,^ 2015) of all gas stations (labelled by the variable *gid*) with 1,888,507 observations. Fig. 1 shows the daily Brent price and daily median value of Diesel price (before taxes).^1^

The second file (*geocoordinates.csv*) includes a cross section with the 8080 gas stations that provide information of the brand, the longitude and latitude coordinates for each gas station and for the 7 Spanish refineries. Table 1 reports the number of gas stations by brand (and strategic groups) and price quotes included in *panel_gas.csv*.^2^

**Table 1:** Number of stations and observations in the database.

	Stations (gid)	Price quotes
Repsol	2930	657,376
Cepsa	1175	267,341
BP	348	79,614
Galp	539	129,001
Shell	309	75,618

Rest of wholesale operators	416	100,196
Independent brands	221	55,706
Low-cost brands	156	38,946
Supermarket brands	277	66,540
Rest of gas stations	1709	417,059
Total	8080	1,888,507

We provide a dofile in STATA (“*panel_gas_G&M.do*”) that includes instructions to identify the three nearest gas stations and their brands, as well as the distance to each of them. Also, it calculates the distance to the nearest refinery and its brand. Finally, we include a dofile (*descriptive.do*) that let to generate tables and graphs included in this article.

## 2 Experimental design, materials, and methods

Our raw data contains the fuel prices of gas stations that operate in Spain. Since 2007, all gas stations have been required to send their fuel prices to the Ministry of Industry, Energy and Tourisms every Monday or whenever they change prices. These prices are then posted on the Website: http://geoportalgasolineras.es. From this site we collect daily price data for retail diesel from 18 August 2014 to 15 June 2015, the location (province plus longitude and latitude). We exclude from our data set those gas stations that operate on the Canary and Balearic Islands, as well as gas cooperatives and other stations that do not sell to the public. The data was collected before midnight, if any station changes the price more than once in a given day, the price collected would be the last price of the day (see González and Moral [2] for more details).

We describe the variables included in the two data files shared with this article in Tables A1 and A2 available in the Appendix.

## Conflict of Interest

The authors declare that they have no known competing financial interests or personal relationships that could have appeared to influence the work reported in this paper.

## Figures and Tables

**Table A.1:** Description of the variables in *panel_gas.csv*.

Name of the variable	Variable description
*gid*	Identifier of the gas station
*t*	Time (in days)
*province*	Variable that identifies the province in which the gas station is located
*Brand*	Name of the brand.
*brand_groups*	Brands grouped reported in Table 1.
*day*	Day of the month 1–31
*month*	Month
*year*	Year: 2014, 2015
*Price_posted*	Posted Price reported by the gas station (original). In € cents per liter.
*Price_prevat*	Posted price net of VAT tax: $Price\_prevat=\frac{Price\_Posted}{\left(1-VAT\right)}$. In € cents per liter. The Value Added Tax (VAT) is 21%.
*Price_pretax*	Posted price net of all taxes is obtained according with this formula: $Price_{pretax}=\frac{Price\_Posted}{\left(1-VAT\right)}-TPP$. Where TPP=TPPgeneral+TPPespecial+TPPregional. Table A3 shows the values of each component of this tax. Information about taxes applied during the sample period can be obtained from the Spanish Ministry of Economy's Tax Office: https://www.agenciatributaria.es/static_files/AEAT/Aduanas/Contenidos_Privados/Impuestos_especiales/Estudio_relativo_2014/IIEE201404.pdf
*Pbrent*	Brent price. It refers to the Europe Brent spot price (FOB, in USD per barrel; obtained from https://www.eia.gov). The official exchange rate is from the European Central Bank: https://www.ecb.europa.eu/stats. One barrel is equal to 159 L, and Brent prices are given in € cents per liter.

**Table A.2:** Description of the variables included in: geocoordinates.csv.

Name of the variable	Description
*gid*	Identifier of the gas station
*Brand*	Brand of the gas station
*glat*	Latitude gas station
*glon*	Longitude gas station
*glan_r1… glan_r7*	Latitude refinery 1 to 7^a^
*glon_r1… glon r_7*	Longitude refinery 1 to 7^a^

a Spanish Refineries: Company and location: **r1**: Repsol. Puertollano (Ciudad Real); **r2**: Cepsa. San Roque (Cádiz); **r3**: Cepsa. La Rábida (Huelva); **r4**: BP. Castellón; **r5**: Repsol. Tarragona; **r6**: Repsol. Bilbao; **r7**: Repsol: A Coruña.

**Table A.3:** Fuel taxes in Spain.

VAT	Taxes on Petroleum Products (TPP) (€ cents per liter)		
	TPP General	TPP Special	TPP Regional^a^
21%	30.7	2.4	0–4.8

a The TPP regional in 2015 was of 0 for Aragón, Cantabria, Galicia, Madrid, Navarra, Rioja and País Vasco. The Autonomous Communities that support the maximum amount of 4.8 were: Andalucía, Valencia, Castilla la Mancha, Cataluña, Extremadura and Murcia. Castilla-León and Asturias imposed an amount of 1.6 and 4 € cents per liter respectively.

**Figure A1:**
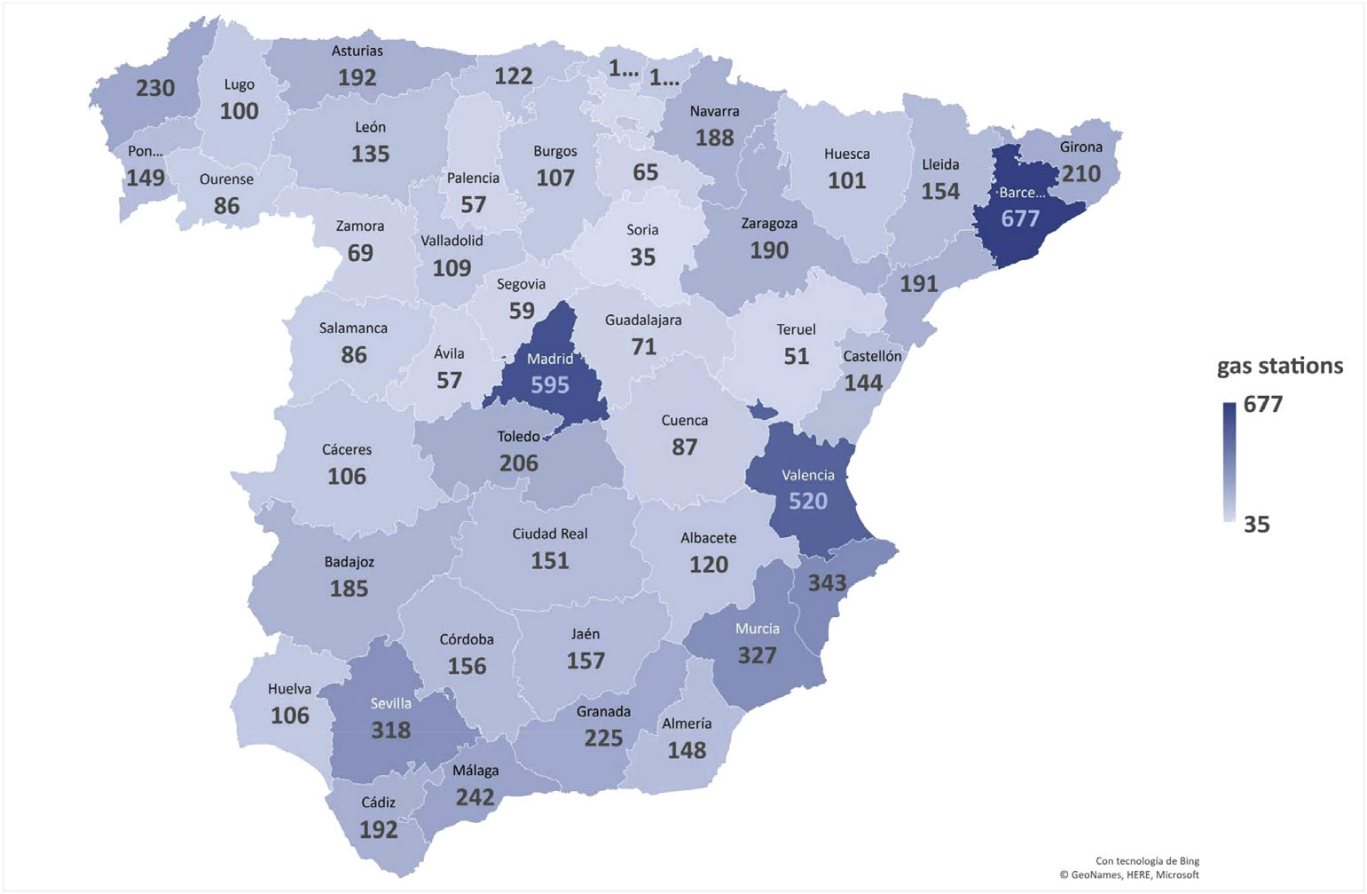
Distribution of gas stations by provinces.

## Footnotes

1 Balaguer and Ripollés [1] share in this journal a data base of gas stations located in Madrid and Barcelona over the period from June 2010 to November 2012.

2 Figure A1 shows the number of gas stations by provinces included in the sample. The provinces of Madrid, Barcelona and Valencia concentrate around one third of all gas stations in Spain.
